# Conservatism Gets Funded? A Field Experiment on the Role of Negative Information in Novel Project Evaluation

**DOI:** 10.1287/mnsc.2021.4107

**Published:** 2021-10-28

**Authors:** Jacqueline N. Lanei, Misha Teplitskiy, Gary Gray, Hardeep Ranu, Michael Menietti, Eva Guinan, Karim R. Lakhani

**Affiliations:** a Harvard Business School, Boston, Massachusetts 02163; b Laboratory for Innovation Science at Harvard, Boston, Massachusetts 02134; c University of Michigan School of Information, Ann Arbor, Michigan 48109; d Harvard Medical School, Boston, Massachusetts 02115; e Dana-Farber Cancer Institute, Boston, Massachusetts 02215

**Keywords:** project evaluation, innovation, knowledge frontier, information sharing, negativity bias

## Abstract

The evaluation and selection of novel projects lies at the heart of scientific and technological innovation, and yet there are persistent concerns about bias, such as conservatism. This paper investigates the role that the format of evaluation, specifically information sharing among expert evaluators, plays in generating conservative decisions. We executed two field experiments in two separate grant-funding opportunities at a leading research university, mobilizing 369 evaluators from seven universities to evaluate 97 projects, resulting in 761 proposal-evaluation pairs and more than $250,000 in awards. We exogenously varied the relative valence (positive and negative) of others’ scores and measured how exposures to higher and lower scores affect the focal evaluator’s propensity to change their initial score. We found causal evidence of a negativity bias, where evaluators *lower* their scores by more points after seeing scores more *critical* than their own rather than *raise* them after seeing more *favorable* scores. Qualitative coding of the evaluators’ justifications for score changes reveals that exposures to lower scores were associated with greater attention to uncovering weaknesses, whereas exposures to neutral or higher scores were associated with increased emphasis on nonevaluation criteria, such as confidence in one’s judgment. The greater power of negative information suggests that information sharing among expert evaluators can lead to more conservative allocation decisions that favor protecting against failure rather than maximizing success.

## 1. Introduction

The annals of business strategy are full of case studies of companies and organizations “squandering good ideas” and failing to innovate (Harford 2018). Famous examples include Kodak not embracing digital photography, Nokia and others unable to shift the mobile phone from a product to a platform, and, most recently, traditional auto manufacturers’ reluctance to invest in electric vehicle technologies (Lagerstedt 2018). The economics and management literature has devoted considerable attention to explaining these failures, including demand-side strategies of disruption (Christensen 2013), supply-side strategies of architectural innovation (Henderson and Clark 1990), industry life-cycle shifts (Tushman and Anderson 1986, Utterback 1994, Klepper 1996), and lack of internal incentives for organizational ambidexterity (O’Reilly and Tushman 2013). Explaining failure, however, is a perilous task, as the number of potential explanations is high, but the opportunities for randomized controlled trials in organizations are very limited. Regardless of the possible strategic explanations for why firms fail to innovate, common to almost all of them is a selection process where novel projects are presented to panels of experts and a deliberative process is undertaken to either accept or reject further efforts in the novel direction (Criscuolo et al. 2017, Azoulay and Li 2020).

Many scholars and practitioners engaged in strategy and innovation research have identified a number of biases in project evaluation and selection processes (Boudreau et al. 2016, Li 2017, Tomkins et al. 2017, Pier et al. 2018), particularly in a direction that favors conservative or risk-averse projects (Galasso and Simcoe 2011, Hirshleifer et al. 2012, Nicholson and Ioannidis 2012, Boudreau et al. 2016, Criscuolo et al. 2017). Although biases in project selection are not entirely surprising, given the challenge of identifying the long-run potential of complex and uncertain ideas with high failure rates (Arrow 2011, Azoulay and Li 2020, Lane et al. 2021, Scott et al. 2020), it is critical to identify the sources of potential biases due to the trillions of dollars spent each year on funding new projects (Li and Agha 2015). In this paper, we investigate the causal role of others' negative opinions of a project in supplanting one's prior positive opinions and, thus, eliminating the project from further consideration.

We focus on the evaluation and selection of novel projects, where evaluation by a panel of experts is often considered the gold standard of assessing project quality (Li and Agha 2015, Boudreau et al. 2016, Criscuolo et al. 2017). A central question in this format of evaluation is how to best aggregate information from multiple experts (Csaszar and Eggers 2013). Information sharing among experts is widely considered beneficial for decision making, and organizations seek to enable idea exchange when feasible (Thomas-Hunt et al. 2003, Cummings 2004). In academic science, reviewers report that seeing others’ opinions helps them evaluate more effectively (Bradford 2017) and believe that discussions enable “the cream” to rise to the top (Lamont 2009). In practice, academic journals have also begun to implement peer-review processes that enable information sharing among evaluators. For example, *Science* has recently introduced *cross-review* into their peer-review process, where evaluators have the opportunity to read each others’ reviews of a manuscript and update them before submitting to the editor (Bradford 2017). However, to date, there has been little systematic evidence on how such information sharing among expert evaluators changes their initial opinions and how such changes may lead to more conservative selection decisions of novel projects.

To better understand how expert evaluators respond to information from other experts, we executed two field experiments in the evaluation of innovative scientific proposals, intervening in the information that is shared between reviewers after they give their initial, independent scores and before they submit their final, possibly revised, scores. Both experiments focused on the valence (positive or negative) of other reviewers’ scores relative to the score of a focal evaluator. Prior research has suggested that people tend to devote more attention and information-processing resources to negative information than to positive information of equal intensity (Peeters and Czapinski 1990, Ito et al. 1998, Rozin and Royzman 2001). This tendency may be heightened in the context of academic reviewing, where experts may have reputation concerns of appearing too lenient (Starbuck 2003, Miller 2006). Thus, we hypothesized that critical scores have a greater influence on evaluators’ judgments than complimentary scores of comparable magnitude.

A critical challenge of this type of research is that the opinions of others that focal evaluators encounter may be associated with the characteristics of proposals or evaluators. To resolve this identification issue, we exogenously expose evaluators to other reviewers’ opinions. We collaborated with the administrators of a research-intensive U.S. medical school to conduct two field experiments based on modifying the details of two research-award processes to make experimental comparisons. We worked closely with the executives of the research organization to manage, administer, and execute the details of the awards, including running the evaluation process. Collectively, our experiments mobilized 369 evaluators from seven universities to evaluate 97 projects, resulting in 761 proposal-evaluation pairs and more than $250,000 in awards. We exogenously varied the relative valence of other reviewers’ scores to which the evaluators were exposed. In the second experiment, we also collected confidential comments from the evaluators explaining their scores. We performed qualitative content coding analysis and topic modeling on these comments to shed light on potential mechanisms for why evaluators chose to update their scores after being exposed to the information treatments.

In both independent experiments, we find evidence of negativity bias, where evaluators lower their original scores of research proposals by more points when the other experts gave “lower scores,” compared with proposals where the other experts gave “higher scores” of comparative magnitude. This negativity bias was confirmed by the qualitative coding of the evaluators’ comments, which revealed that the evaluators expended greater effort on the evaluation task to find additional limitations, weaknesses, and problems with the proposal after they were exposed to lower scores. Neutral and higher scores were more likely to be associated with increased confidence in one’s initial judgment and motivations to be consistent with other reviewers, and corresponded to less information processing of the evaluative criteria. The need to find more demerits when evaluators learned that their original scores were better than the other reviewers’ suggests that evaluators tend to systematically focus on the weaknesses of the proposed work, rather than its strengths.

Our paper makes several contributions to understanding expert evaluation of novel projects. First, and most importantly, our findings show that evaluators place more weight on negative information and suggest that information sharing may promote, possibly unintentionally, proposals with the fewest weaknesses over those with the best balance of strengths and weaknesses. In other words, information sharing may favor selection of more conservative (or conventional) research portfolios. The relationship between information sharing and conservative selections has potentially economy-wide implications. Expert evaluation panels are used across economic domains, from academic science (Pier et al. 2018) to boards (Zhu 2013) to industrial research and development (Criscuolo et al. 2017), and stakeholders often perceive that evaluators prefer conservative ideas (Nicholson and Ioannidis 2012). However, hitherto, the connection between evaluation format and outcomes had been unclear. This study provides an important step in explaining the connection. Our work also opens new avenues for strategy research by highlighting the salience of selection processes as having a sizeable impact on how executives choose their strategies. Lastly, our work presents an interventional research design that is a novel departure from other studies of the evaluation process. Our effects are not dependent on the underlying quality of the proposal, the attributes of the evaluators, or associations between proposals and evaluators.

## 2. Selecting Novel Projects Through Expert Evaluation and Information Sharing

A persistent concern in the evaluation and selection of novel projects is the tendency of decision makers to favor conservative projects (Nicholson and Ioannidis 2012, Wagner and Alexander 2013, Criscuolo et al. 2017). Here, we take conservative projects to broadly mean those with few weaknesses (“safe” projects) rather than the best balance of strengths to weaknesses (for example “high risk, high reward” projects). The concern of a conservative bias is long-standing in academic peer review (Roy 1985), but direct, rigorous study of whether and how the bias arises is missing. Below, we consider how conservatism may be related to the format of how novel projects are evaluated and, in particular, information sharing among expert evaluators.

Prior empirical work and formal models both suggest that information sharing can improve decision quality, increasing reliability and decreasing bias, particularly when it incorporates structured processes (Dalkey 1969, Bartunek and Murninghan 1984, Gigone and Hastie 1993, Okhuysen and Eisenhardt 2002, Csaszar and Eggers 2013, Wagner and Alexander 2013). However, the formal models face two challenges: What objective function do the individuals seek to maximize, and how do they weigh others’ opinions? For example, in a simple Bayesian model, an individual evaluator seeks to maximize the accuracy by first estimating some parameter, say, the long-term value of a project, and updates it based on others’ opinions weighted by their skills (Gelman et al. 2004). However, in practice, both the objective function and how others’ skills are perceived can be more complex. Evaluators may seek objectives unrelated to quality, such as promoting fairness or equity (Fehr et al. 2006), furthering one’s reputation (Levy 2007), or simply coordinating around some “best” proposal, regardless of private opinions about its quality (Correll et al. 2017, Sharkey and Kovács 2018). Hence, understanding the implications of information exposure in the field requires determining empirically what type of information evaluators attend to and integrate into their own beliefs. In the remainder of this section, we focus on the valence of external information, specifically, how negative and positive information from others may differentially affect an individual’s receptivity to it.

### 2.1. Valence of Others’ Information

Research in judgment and decision making suggests that people generally weigh negative information more highly than positive information of comparative magnitude (Peeters and Czapinski 1990, Ito et al. 1998, Rozin and Royzman 2001). This effect, called negativity bias, may arise through a variety of processes, from adaptive pressures during evolution to negative information’s greater veracity, diagnosticity, and contagiousness (Peeters and Czapinski 1990, Taylor 1991, Baumeister et al. 2001, Rozin and Royzman 2001, Hilbig 2009). In the context of evaluating novel projects, negative and positive information arises most obviously in one’s assessment of the weaknesses (negatives) and strengths (positives) of an idea.

Moving to our context of academic science, the grant-proposal review process is often performed collaboratively (Pier et al. 2018). During the review process, other reviewers’ evaluations offer sources of positive and negative information and have the potential to invite reconsideration of one’s own evaluation. Hence, the tendency to place greater weight on negative information is likely to direct scientists’ attention toward a proposal’s weaknesses. In addition to the general negativity bias, three arguments point to the greater strength of negative information in evaluating novel scientific ideas: association between criticism and expertise, retrospective versus prospective evaluation, and reputational concerns.

First, Robert Merton famously argued that what makes science successful is the norm of “organized skepticism,” according to which all claims are approached with intense scrutiny and detached skepticism (Merton 1973). To this end, scientists are often trained to debate the merits of different theories and methods, which may result in an emphasis on the potential flaws in proposed work (Starbuck 2003, Miller 2006). Empirical work supports this view by showing a strong connection between expertise and criticism (Gallo et al. 2016, Mollick and Nanda 2016), with scientists assigning systematically lower scores to proposals when they know more about the domain (Boudreau et al. 2016). Upon seeing lower scores, focal evaluators may infer that those scores come from more expert or diligent evaluators and allocate more cognitive effort to searching for overlooked weaknesses.

Second, the progression of scientific ideas from conception to implementation is a lengthy and complex process involving several stages of evaluation (Boudreau and Lakhani 2015). Prospective evaluation of novel work to be done is likely to differ from retrospective evaluation of already, or mostly, completed work in the information available to the evaluator. In retrospective evaluation, such as manuscript peer review, the strengths of the work are largely known and, ideally, articulated by the authors; the evaluators’ role is to identify the weaknesses (Fiske and Fogg 1990). In contrast, prospective evaluation, such as in grant-proposal review, occurs in a very different information environment, in which the true underlying strengths and weaknesses of the work are not fully known to either authors or evaluators. Such evaluation is largely about forecasting the future, and a focus on the negatives (compared with a more balanced approach) may be suboptimal (Åstebro and Elhedhli 2006). Yet, evaluators may, nevertheless, bring the typical focus on the negatives to prospective evaluations as well (Gallo et al. 2016).

Third, the collaborative nature of prospective evaluations also creates opportunity for reputational concerns. Learning that others found more problems in an idea than oneself may threaten one’s self-concept as an expert (Amabile and Glazebrook 1982), which to scientists is key (Lamont 2009). Consequently, evaluators may deploy criticism for impression management. Moreover, a reputational concern to appear accurate, knowledgeable, and thereby critical may play an important role in how evaluators update their evaluations (Amabile 1983). Taken altogether, we hypothesize:

#### Hypothesis (“Negativity Bias”).

Evaluators lower their scores by more points in response to negative information than raise them in response to positive information of comparable magnitude.

## 3. Research Design

**Figure 1.**
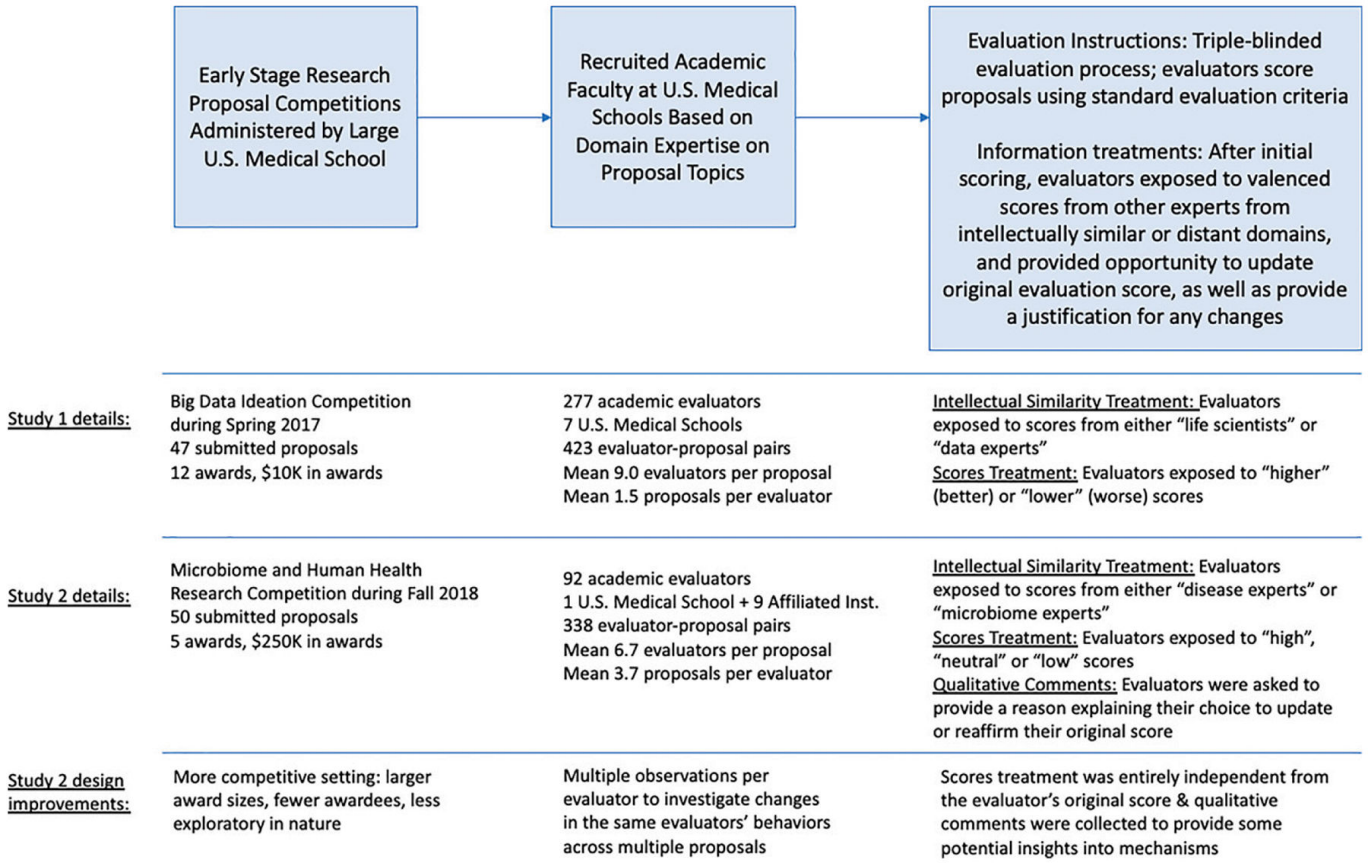
Overview of Research Setting, Evaluator Recruitment/Selection, and Treatment Conditions *Note.* Inst., institutions.

In this section, we describe the key aspects of the research design—namely, the research setting, recruitment of evaluators, and treatment conditions—for both studies in parallel. Figure 1 provides a summary of these aspects of the research design and also highlights the design improvements in study 2 that were informed by the lessons learned from study 1. We conclude the section by describing the main variables and our empirical estimation strategy.

### 3.1. Research Setting

As shown in Figure 1, both studies leveraged translational research-proposal competitions administered by a large U.S. medical school, where our research team cooperated with the award administrators to intervene in the evaluation process. Translational (“bench to bedside”) research is the process of applying discoveries from the laboratory and preclinical studies to the development of techniques that can address critical patient needs in the clinic (Rubio et al. 2010).

Study 1 was a translational research ideation competition that called for proposals of computational solutions to human health problems. Specifically, the call asked for applicants to:

Briefly define (in three pages or less) a problem that could benefit from a computational analysis and characterize the type or source of data.

The competition was advertised nationwide by the U.S. National Institutes of Health-funded Clinical and Translational Science Awards (CTSA) Centers, open to the public, and applications were accepted from 2017-06-15 to 2017-07-13.

The call yielded 47 completed proposals. The vast majority of applicants were faculty and research staff at U.S. hospitals. Clinical application areas varied widely, from genomics and oncology to pregnancy and psychiatry. Twelve awards were given to proposals with the highest average scores (eight awards of $1,000 and four awards of $500). Evaluators were aware of the award size and that multiple projects would be selected. Submitters were aware that their proposals might be considered as the basis for future requests for proposals for sizeable research funding.

Study 2 was a translational research proposal competition on Microbiome in Human Health and Disease. The competition called for proposals that promote a greater understanding of the role(s) micro-biomes play in maintenance of normal human physiology and in the manifestation and treatment of human disease. Specifically, the call asked for applicants to:

Think broadly about the interactions between micro-biomes and human physiology and ecology in formulating their proposals.

The competition was open to members with a university appointment, and applications were accepted from October 18, 2018 to November 20, 2018, with award decisions announced in January 2019. Clinical application areas varied widely, including surgery, cardiology, oncology, and Alzheimer’s disease. The call yielded 50 completed proposals. Five awards of up to $50,000, for a total of $250,000 in funding, were given to proposals with the highest average scores.

Hence, although both studies leveraged translational research-proposal competitions—which provided a controlled environment to essentially replicate the information treatments in study 1 in study 2, the larger and more competitive award setting of study 2, combined with the less exploratory nature of the proposal applications, was more representative of typical research-award competitions in biomedicine (Azoulay and Li 2020), which also enabled us to examine whether and to what extent the evaluators’ behaviors would replicate in a higher-stakes evaluation and selection process.

### 3.2. Evaluator Recruitment and Selection

As illustrated in Figure 1, we recruited faculty members from multiple U.S. medical schools to be evaluators, based on their domain expertise in the proposal topic areas. These proposal topic areas were determined by administrators, as part of the standard process for recruiting potential evaluators. To recruit internal reviewers, the award administrators used a university-wide database to identify researchers by topic area using their concept areas, Medical Subject Headings (MESH) terms, and recent publications. External evaluators were identified by using the CTSA External Reviewers Exchange Consortium (CEREC). The proposals were posted to the CEREC Central web-based tracking system, and staff at the other hubs located evaluators whose expertise matched the topics of the proposals. One benefit of this standardized process is that the evaluators did not self-select the number of proposals to review. Rather, the number of proposals reviewed by each evaluator was determined ex ante by the award administrators based on their categorization of the proposal topic areas.

In study 1, there were a total of 277 evaluators from seven U.S. medical schools, for a total of 423 evaluator-proposal pairs. The proposals were grouped by topic (17 topics), with cancer being the largest group (14 proposals). Each proposal was reviewed by a mean of 9.0 evaluators (min = 7, max = 13, standard deviation (s.d.) = 1.5); 71.5% of evaluators completed one review, 14.8% completed two reviews, and 13.7% completed three or more reviews, for a mean of 1.5 proposals per evaluator (min = 1, max = 6, s.d. = 1.06). Because most evaluators conducted just one review, one limitation of study 1 is that we could not collect multiple observations per evaluator under different randomized treatment conditions.

In study 2, a total of 92 evaluators were selected from the sponsoring university and nine affiliated institutions for a total of 338 evaluator-proposal pairs covering 14 proposal topics, with cancer and gut microbiome and disease being the largest groups (eight proposals in each). To examine the same evaluators’ behaviors across different exogenous treatment conditions, we worked closely with the award administrators to assign each recruited evaluator multiple proposals to review, to facilitate multiple observations of the same evaluators. Consequently, each proposal was reviewed by a mean of 6.7 evaluators (min = 3, max = 13, s.d. = 2.61), and each evaluator completed a mean of 3.7 proposals (min = 1, max = 8, s.d. = 2.5). Collectively, we recruited 369 evaluators to evaluate 97 proposals, for a total of 761 evaluator-proposal pairs.

### 3.3. Evaluator Instructions and Treatments

The evaluation process, conducted online, was triple-blinded: Applicants were blinded to the evaluators’ identities, evaluators were blinded to the applicants’ identities, and evaluators were blinded to each others’ identities. Anonymity is a critical feature of our experimental design. In identifiable situations, individuals may choose to adopt or reject others’ opinions according to their credibility (e.g., knowledge and expertise) or status (Dovidio et al. 1998, Bendersky and Hays 2012, Correll et al. 2017). Anonymity mitigates social cues to update scores and isolates informational motives (van Rooyen et al. 1998, Tomkins et al. 2017). Figure A1 in the online appendix provides a screenshot of the evaluator instructions and sample information treatments from study 2, but evaluation procedures were similar in both studies, and differences are discussed below.

Evaluators were asked to score proposals using a similar rubric to that used by the National Institutes of Health, with which they are broadly familiar. Both studies asked evaluators to use the following criteria for scoring the proposals: feasibility, impact, innovation, and expertise (1 = worst to 6 = best in study 1; 1 = worst to 5 = best in study 2), as well as provide an overall scientific merit score (1 = worst, 8 = best in study 1; 1 = worst, 9 = best in study 2). In study 1, evaluators were also asked to rate their confidence in their original evaluation score (1 = lowest, 6 = highest). In study 2, instead of having evaluators rate their confidence in the original evaluation score, we asked them to state whether they would designate a top-3 ranking to the current proposal (conditional on having reviewed three or more proposals). We also asked evaluators to self-identify as either microbiome or disease domain experts. Evaluators in the control condition were simply shown their own scores again and given the opportunity to update. This condition was designed to account for the possibility that simply giving evaluators the opportunity to update may elicit experimenter demand effects, resulting in updating behavior that is coincidental to, not caused by, the external information.

After recording all scores, evaluators in the treatment condition proceeded to a screen in which they observed their scores next to artificial scores attributed to other reviewers who were either from intellectually similar or distant domains.^1^ In study 1, the “Other reviewers” were randomly assigned to either *scientists with MESH terms like yours* or *data science researchers.* The first variant of Other reviewers signals that other reviewers are life scientists, whereas the second variant signals that the other reviewers are data experts that apply their skills to human health problems. In study 2, the Other reviewers were randomly assigned to be either *disease-specific experts* or *microbiome experts.* The first variant indicated that the other reviewers were disease (human health) researchers who may or may not have worked with microbiome to advance understanding of diseases, whereas the second variant indicated that the other reviewers were microbiome researchers who worked with microbiome to understand its role in maintenance of human physiology.

The scores were presented in a range (e.g., “2–5” or “7–9”) to appear as coming from multiple reviewers (although we did not indicate how many). We chose this presentation format because previous research has shown that the degree to which individuals utilize external information increases with the number of independent information sources and their unanimity (Mannes 2009). After viewing the (artificial) scores, evaluators were given an opportunity to update their own scores. Below, we describe the information treatments in more detail.^2^

#### 3.3.1. Study 1 Treatment Conditions.

Table 1 shows that 244 evaluators were assigned to the treatment conditions in study 1, with each evaluator completing a mean of 1.59 reviews (min = 1, max = 6, s.d. = 1.05, *n* = 244) and 34 evaluators assigned to the control condition, with each evaluator completing a mean of 1.0 review (min = 1, max = 2, s.d. = 0, *n* = 34). Moreover, Table A1 in the online appendix shows that each proposal in the treatment condition was evaluated by a mean of 8.28 evaluators (min = 5, max = 10, s.d. = 1.33, *n* = 47) and by a mean of 1.36 evaluators in the control condition (min = 1, max = 5, s.d. = 0.89, *n* = 25).

In study 1, the artificial treatment scores were presented as a range—for example, 2–5—and the range of scores was always directionally two to three points either slightly above or below the initial evaluation score given by the focal evaluator. In other words, evaluators in the treatment condition were always exposed to *relative* feedback, where the opinions (i.e., scores) of the other reviewers were always unanimously different from the subjects in the experiment. Table A2 in the online appendix summarizes how the treatment scores were constructed, relative to the evaluator’s original score, and indicates that only the middle scores, between 3 and 6, were completely randomized to both lower and higher scores, with respect to the original proposal score.

#### 3.3.2. Study 2 Treatment Conditions.

Table 1 shows that 89 evaluators were assigned to the treatment condition in study 2, with each evaluator completing a mean of 3.75 reviews (min = 1, max = 8, s.d. = 2.43, *n* = 89). There were also three evaluators assigned to the small control condition, where each evaluator completed a mean of 1.50 reviews (min = 1, max = 2, s.d. = 0.50, *n* = 3). Table A1 in the online appendix shows that each proposal was reviewed by a mean of 6.68 evaluators (min = 3, max = 13, s.d. = 2.56, *n* = 50) in the treatment condition and by a mean of 2.00 evaluators in the small control condition (min = 2, max = 2, s.d. = 0, *n* = 2). We note that in the design phase of study 2, we focused primarily on the valenced treatment conditions, and not the control condition.

In study 2, we exogenously varied the other reviewers’ scores over the entire range of possible scores and constructed three score ranges, “1–3,” “4–6,” and “7–9,” that corresponded to “low,” “moderate,” and “high” treatment scores, respectively. In other words, the evaluators were always exposed to *absolute* feedback that was independent of their own initial score, and they were shown scores indicating whether the other reviewers gave the same proposal low, moderate, or high scores. This meant that evaluators could be exposed to treatment scores that could be directionally lower, higher, or within the same range as the other reviewers. Table A3 in the online appendix depicts the number of evaluator-proposal pairs in each valenced treatment and control condition and indicates whether the treatments came from intellectually close or distant reviewers.

#### 3.3.3. Qualitative Comments.

One key aspect of the design in study 2 was to collect qualitative comments of the evaluators’ reasons for updating their original scores. To this end, we worked closely with the award administrators to execute this nonstandard question within the evaluation form. After evaluators were provided the opportunity to update their scores, there was a text box on the same page of the screen that asked them to *please explain* why they updated their overall score of the current proposal (see Figure A1 in the online appendix).

### 3.4. Main Variables

#### 3.4.1. Dependent Variables.

Our main dependent variable, *Change in evaluation score,* measures the difference between the updated score (after exposure to the treatment scores) and the original score. Figure A2 in the online appendix shows the distribution of score updates by treatment valence for study 1 (left) and study 2 (right) and shows that evaluators updated their scores in the direction of the other reviewers’ scores more than 99% of the time (there were only two cases of score updating in the opposing direction).

#### 3.4.2. Independent Variables.

Our main independent variable, *Treatment score valence,* corresponds to the direction of the treatment scores and was coded as a categorical variable indicating whether the treatment scores from the other reviewers were strictly lower than the evaluator’s original score, strictly higher than the evaluator’s original score, or within the same range as the evaluator’s original score:

Treatmentscoresvalence={lowertreatmentscoresif[treatmentscoresrange]<originalscorehighertreatmentscoresif[treatmentscoresrange]>originalscoreneutraltreatmentscores,otherwise.}

For example, if an evaluator gave a proposal an initial score of 5 and was exposed to treatment scores of 1–3, then the categorical variable, *Treatment scores valence* would take the value of *lower treatment scores* because 1–3 is less than 5; however, if the same evaluator were instead exposed to treatment scores of 7–9, then *Treatment scores valence* would take the value of *higher treatment scores* because 7–9 is higher than 5; finally, if the same evaluator were exposed to treatment scores in the same range as the evaluator’s own score, then *Treatment scores valence* would take the value of *neutral treatment scores.* Table 2 shows the distribution of treatments by the original score for each study and the valence of the treatment scores.

#### 3.4.3. Other Variables and Controls.

The analysis strategy relies most critically on the research design’s randomization of valenced treatment scores and exploitation of multiple observations per proposal and evaluator. We use dummy variables for evaluators and proposals to control for time-invariant unobserved evaluator and proposal characteristics. We also use a number of evaluator and evaluator-proposal covariates to examine descriptively when evaluators are more or less likely to change their scores. We control for *Original score,* the gender and the status of an evaluator with *Female* and *High rank* (= 1 if associate or full professor), and the evaluator’s self-reported *Expertise* on the proposal topic.^3^ Lastly, we control for *Intellectual distance*, which is equal to one if the evaluator and other reviewers were from different fields. In study 1, a third-party expert coded each evaluator’s expertise as being either in the life sciences or data science. In the second study, we used the evaluator’s self-identified expertise as microbiome or disease expert to code whether the evaluators and the Other reviewers were intellectually close or distant.

### 3.5. Estimation Approach

We performed ordinary least squares regressions on the pooled studies to estimate the relationships between the likelihood and size of evaluators’ updating behaviors on the treatment effect of being exposed to relative and absolute feedback on other reviewers’ proposal scores. Our main analysis focuses on comparing the effects of valenced feedback on the evaluators’ updating behaviors, as none of the evaluators in the control condition updated their scores. Our simplest model includes the *Treatment scores valence* variable only, for each evaluator-proposal pair, {*i*,*j*}. We then add controls for evaluator-proposal attributes (intellectual distance and expertise) and evaluator attributes (gender and high rank), as well as original score fixed effects (*γ_ij_*). Because the treatment scores were not independent of the original evaluation score, controlling for the original score was critical to allow for comparisons between evaluators that gave the same original score, but were randomly assigned to lower, neutral, or higher treatment scores.

The main model for the relative feedback exposures is presented in Equation (1):

(1) $$Change\;in\;evaluation\;score_{ij}\;\;=\beta_{0}+\beta_{1}Treatment\;scores\;valence_{ij}\;\;+\beta_{2}Evaluator\;Proposal\;attributes_{ij}\;\;+\beta_{3}Evaluator\;attributes_{i}+\gamma_{ij}+\varepsilon_{ij}.$$

To examine within-evaluator differences, we add evaluator fixed effects (*γ_i_*) to (1) for the subset of evaluators who reviewed more than one proposal.

As a robustness check to improve identification of the effect of *Treatment scores valence*, we restrict our sample to only those observations that were eligible to receive both higher and lower treatment scores—that is, reviews where the original score was in the “middle” of the scoring range. Table 2 shows that this subset corresponds to original scores of 3–6 in study 1 and 4–6 in study 2, for a total of 274 and 156 evaluator-proposal pairs, respectively, and 430 evaluator-proposal pairs across the two studies.

## 4. Results from Quantitative Analyses

Table 3 presents the covariate balance checks for the 723 evaluator-proposal pairs in the treatment condition that were exogenously exposed to the valenced score treatments. As expected, the covariates are not associated with treatment assignments, except for the original score (as only middle scores were exogenously exposed to both lower and higher scores from other reviewers). Table 4 presents the correlation table of the main variables.

Table 5 presents the main regression results examining the estimated relationships between the evaluators’ score-updating behaviors and treatment-scores valence. Models 1–4 present the regression results on the full sample of 723 evaluator-proposal pairs from the pooled studies, and models 5–8 present the regression results on the restricted sample of middle scores of 430 evaluator-proposal pairs that were exogenously exposed to both lower and higher treatment scores. In all models, we use neutral treatment scores as the baseline condition, in order to make comparisons between the magnitude and direction of evaluation-score updates from exposures to lower and higher treatment scores.

Turning to the full sample, we begin with the most straightforward regression of evaluators’ *Change in evaluation score* on *Treatment scores valence*. We observe in model 1 that, relative to neutral treatment scores, evaluators exposed to lower treatment scores lowered their original scores by 0.759 points (standard error (s.e.) = 0.055) and raised them by a smaller magnitude of 0.449 points (s.e. = 0.0499) after being exposed to higher scores. We add evaluator and evaluator-proposal covariates in model 2. The coefficients for lower and higher treatment scores remain stable and significant (model 2: lower: −0.756, s.e. = 0.0557; higher: 0.452, s.e. = 0.0523). Model 3 then adds original score fixed effects to allow for comparisons between evaluators who gave the same original score. The coefficients for lower and higher treatment scores still remain stable and significant (model 3: lower: −0.753, s.e. = 0.0580; higher: 0.434, s.e. = 0.0581).

**Figure 2.**
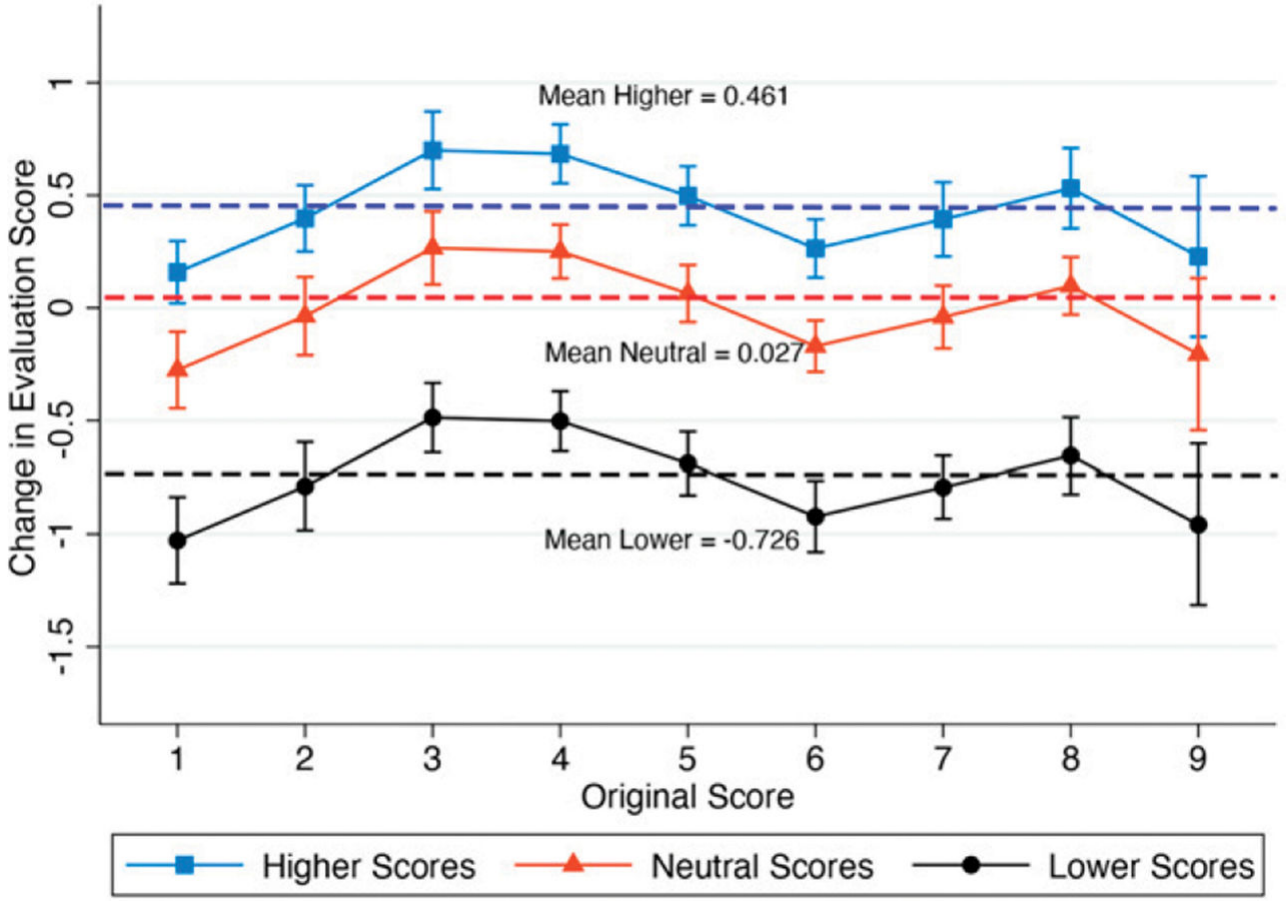
Margins Plot of Change in Evaluation Score and Treatment Scores Valence by Original Score with 95% CIs

To better understand these estimated relationships by original score, Figure 2 plots the change in evaluation score with 95% confidence intervals (CIs) by the valenced treatment scores and original evaluation score using the coefficients from model 3. In Figure 2, we observe that evaluators lowered their original scores by 0.726 points, on average, after exposures to lower scores and raised them by a smaller magnitude of 0.461 points, on average, after exposures to higher scores. In contrast, exposures to neutral treatment scores led to minimal score-updating behaviors of 0.027 points, on average. Thus, Figure 2 provides complementary evidence indicating that lower treatment scores led to larger updates in a negative direction, compared to higher treatment scores of comparative magnitude. Lastly, in model 4, we add evaluator fixed effects (FE) for the subset of evaluators that reviewed more than one proposal to examine within evaluator differences. The coefficients for lower and higher treatment scores once again remain consistent (model 4: lower: −0.867, s.e. = 0.103; higher: 0.527, s.e. = 0.106). Taken together, this suggests that evaluators lower their scores by a greater magnitude after exposures to lower scores than raise them after exposures to higher scores.^4^

It is important to note the economic significance of these coefficients: In particular, exposures to lower treatment scores resulted in updated scores that were 1.2 points lower than the updated scores treated with higher scores. Given that the mean original score across both studies is 5.1 (Table 4), the resulting difference corresponds to a 23.5% decrease between the updated scores that were treated with lower versus higher scores.

Next, we present the regression results using the restricted sample of middle scores that were exposed to *both* lower and higher treatment scores (see Table 2 and Section 5.3), thereby excluding any tail scores that only received one of the valenced treatment scores. Model 5 shows that, relative to evaluators exposed to neutral treatment scores, evaluators exposed to lower treatment scores lowered their original scores by 0.625 points (s.e. = 0.0816). In contrast, evaluators raised their scores by a smaller magnitude of 0.561 points (s.e. = 0.0635) after exposures to higher scores (compared with the neutral treatment scores baseline). The coefficients for lower and higher treatment scores remain consistent and significant in model 6, which adds evaluator and evaluator-proposal attributes (lower: −0.618, s.e. = 0.0827; higher: 0.567, s.e. = 0.0650); in model 7, which adds the original score FE (lower: −0.663, s.e. = 0.0823; higher: 0.503, s.e. = 0.0701); and, finally, in model 8, which adds evaluator FE for the subset of evaluators who reviewed more than one proposal (lower: −0.911, s.e. = 0.158; higher: 0.512, s.e. = 0.157).

In supplementary analyses, we show that this finding of a negativity bias is consistent with the absolute magnitude of the evaluation-score updates (see Tables A4 and A5 in the online appendix), for each study separately (see Tables A6 and A7 in the online appendix), and with the control condition added (see Table A8 in the online appendix). Altogether, we find robust support for our main hypothesis that evaluators are more likely to lower their scores after being exposed to negative information than raise them in response to positive information, yielding a negativity bias.

## 5. Qualitative Analyses: Content Coding

In study 2, after being assigned the treatment scores, evaluators were asked to explain why they changed (or did not change) their scores (see Figure A1 in the online appendix). The formal analysis of the evaluator comments consisted of three main stages of coding (Lofland and Lofland 1971, Charmaz 2006). We began with open coding, identifying codes based on the evaluators’ written responses about their justifications or reasons provided for adjusting their scores. Examples of open codes included “admit did not see all weaknesses,” “reread proposal and provided reason for changing score,” or “feasible proposal with possible impact.” Next, we grouped open codes in abstract bundles in the second step of axial, more focused coding. These categories evolved as we iterated among the data, emerging themes, and existing literature. Examples of axial codes included “consistent with others” (if evaluators adjusted their score to be more aligned with the other evaluators) and “design and methods” (if evaluators pointed out a strength or weakness about the research design and/or methods). In the third stage, we further explored the relationships among the abstract codes and aggregated them into emergent primary topics. We performed analyses on 286 of the total 321 (89.1%) reviews in the study sample where evaluators were exposed to lower, neutral, or higher scores. Comments were excluded if the evaluator did not provide an explanation for why they chose to update (or not update) their original score (e.g., “N/A,” “no further comments,” or “no update”). Table 6 summarizes the data taxonomy resulting from the analytic process.

### 5.1. Content Coding Results

**Figure 3.**
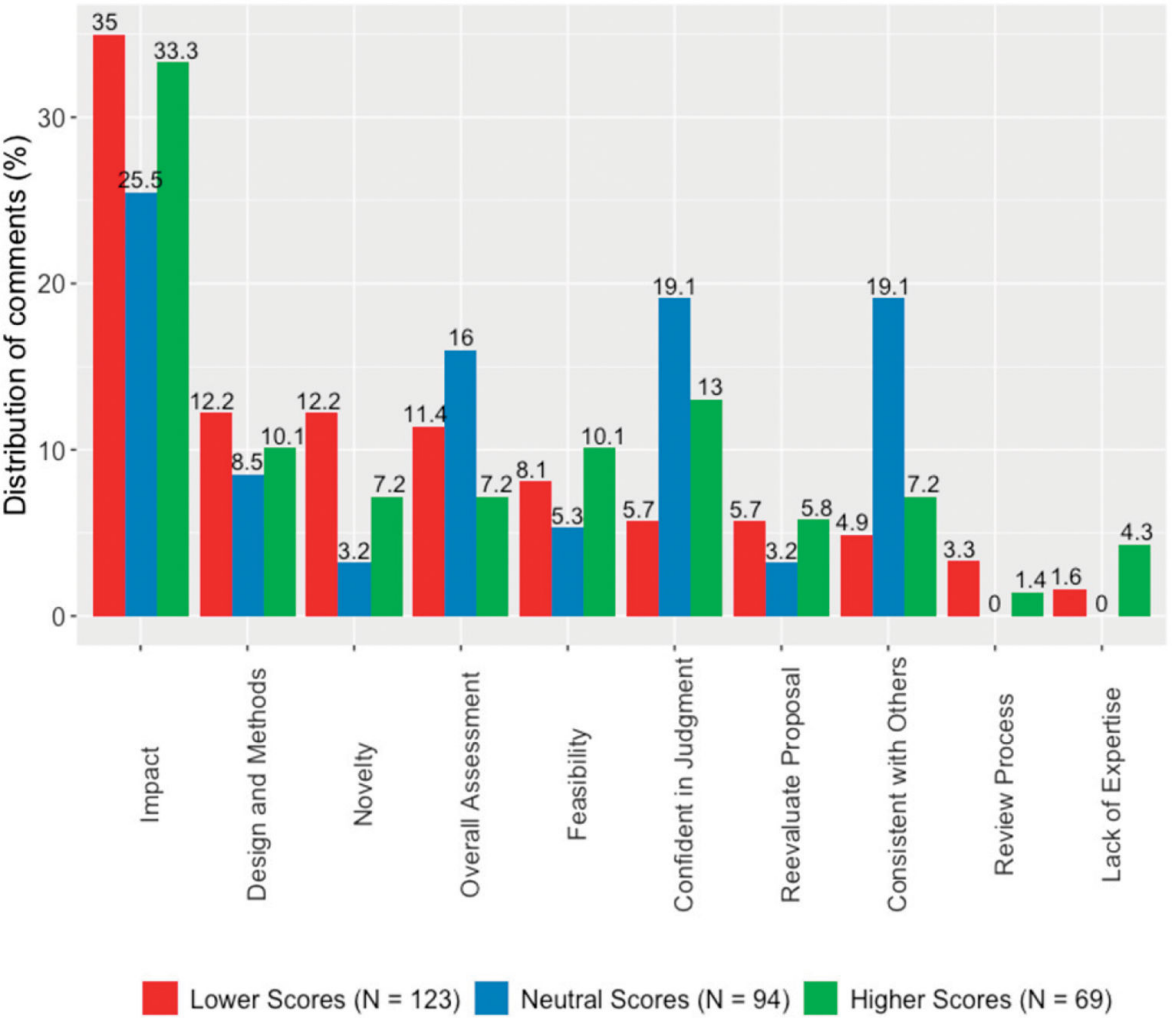
Distribution of Axial Codes by Valenced Treatment Scores (Study 2)

Figure 3 plots the distribution of comments by the valenced treatment-score exposures to lower, neutral, and higher treatment scores according to the axial codes described in Table 6 and ordered by the distribution of coded comments for the lower treatment scores. The remainder of this section focuses on interpreting the distribution of the axial codes to unpack the observed asymmetry in the evaluators’ behaviors.

#### 5.1.1. Unpacking Impact and Treatment-Score Valence: Allocating Attention to Strengths vs. Weaknesses.

When the evaluators were prompted to explain their reasons for updating their scores, *Impact* emerged as a top code for all three treatment-score valences, corresponding to 35.0%, 25.5%, and 33.3% of comments for lower, neutral, and higher treatment scores, respectively. Interestingly, the majority of comments coded as *Impact* was related to critiques or weaknesses of the potential benefit of the project in terms of improved treatments or increased understanding of disease.^5^ Below is a sample comment from evaluator A, who had received a lower score exposure of 1–3 after scoring the proposal a 5. After receiving the other scores, the evaluator changed their score to 3:

[Modifier] is the major factor affecting the gut microbiota. Authors did not explain how they would control the impact of the modifier] as a confounding factor on these two groups of study subjects. In subjects with [targeted syndrome], we will not know whether the gut microbiome alterations could be the cause for this syndrome or another syndrome and/or [modifier] alters the gut microbiota. Clinical impact would be minimal with this project.

Similarly, below is a comment from Evaluator B, who received treatment scores of 4–6 after scoring the proposal a 4. After receiving the other scores, the evaluator did not change their score:

Even if the [modifier 1] and [modifier 2] are part of human diet, the merit is not clear. In addition, up to 4% of [modifier 1] was used in the mouse study, but proposed human study is far less (as % of diet). This condition has not been justified in the animal study.

These accounts exemplify how other reviewers’ scores focused evaluators’ attention on identifying and justifying the weaknesses, limitations, and demerits of the proposal, rather than its strengths.

#### 5.1.2. Lower Treatment Scores and Shifting Attention to the Evaluation Criteria.

Although *Impact* was the most prevalent code, the distribution of the remaining axial codes differed depending on the valence of the treatment. As shown in Figure 3, whereas the next five most common axial codes in the lower-treatment-scores condition corresponded directly to the evaluation task at hand (*Design and Methods, Novelty, Overall Assessment, Feasibility,* and *Reevaluate Proposal*), the rank order of axial codes for the neutral and higher treatment scores conditions were more evenly distributed between codes focusing on both the evaluative and nonevaluative criteria, where the latter refers to comments not *directly* related to the evaluation task, such as agreeing with others. In particular, the second most prevalent axial code for neutral treatment scores was *Confident in judgment* (19.1%) and *Consistent with others* (19.1%), and for higher treatment scores, it was *Confident in judgment* (13.0%).

A few sample comments illustrate this pattern. The first is from evaluator C, who had provided an original score of 4, was exposed to neutral treatment scores of 4–6, and did not update his score postexposure: “I stand by my initial score.” The second is from evaluator D, who had provided an original score of 2 and received higher treatment scores of 4–6 and also chose not to update her score postexposure: “My score is not influenced by other reviewers’ evaluation.” Both of these comments were coded as *Confident in judgment.* A third comment, coded as *Consistent with others*, is from evaluator E, who had provided an original score of 5, was exposed to neutral treatment scores of 4–6, and chose not to update postexposure: “My score is consistent with those of other reviewers. I will stick with it.” These comments contrast substantively to those given for the lower treatment scores, as here, the evaluators demonstrate less desire or to reevaluate their original opinion or justify them to the reader.

**Figure 4.**
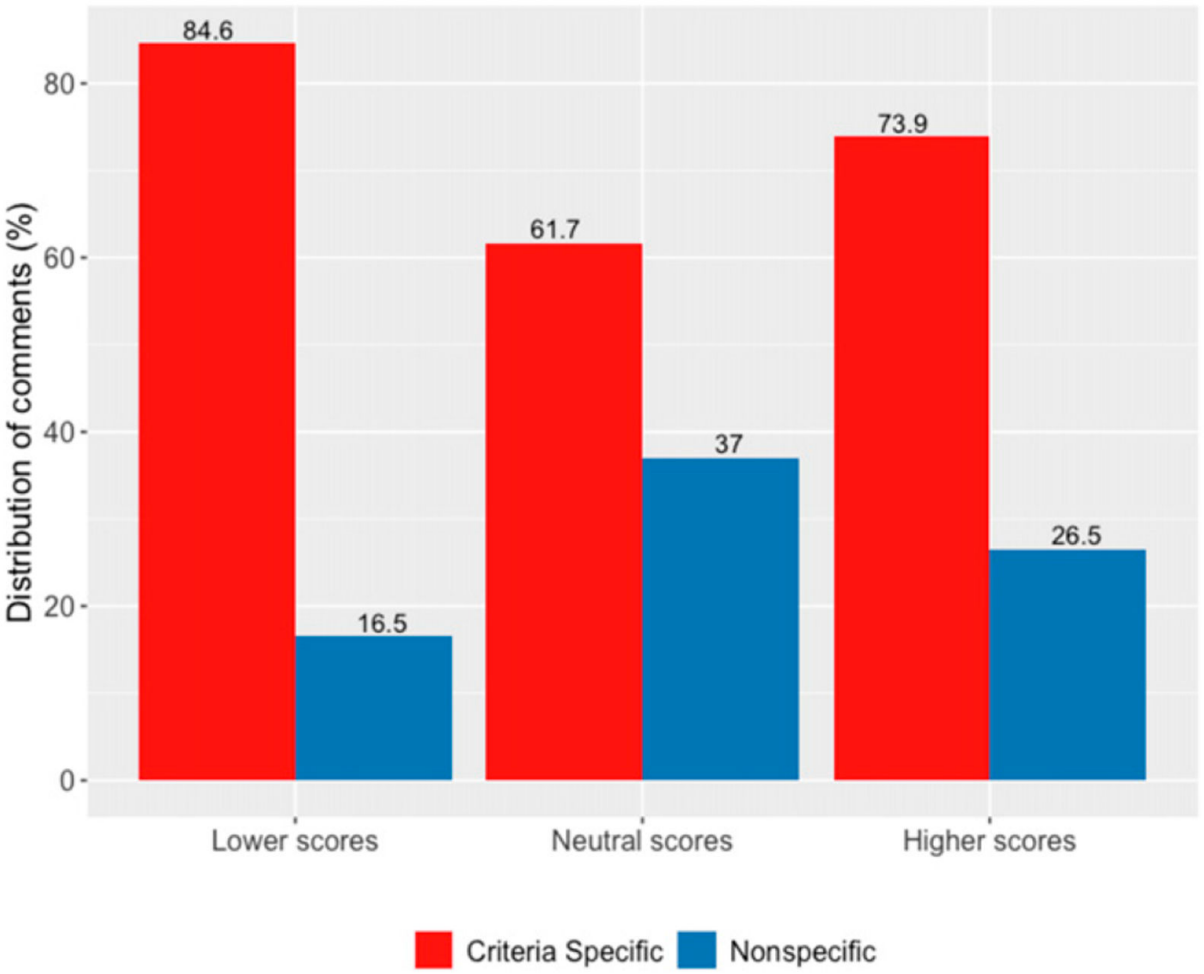
Distribution of Primary Topics by Valenced Treatment Scores (Study 2)

Next, Figure 4 aggregates the axial codes into primary topics and shows the distribution of comments by evaluation-criteria-specific or nonspecific topics by the treatment valences. Whereas 84.6% of comments were coded as criteria-specific in the lower-treatment-scores condition, this compares to 61.7% in the neutral-treatment-scores condition and 73.9% in the higher-treatment-scores conditions, respectively. A two-tailed binomial test indicates that both proportions are significantly different from the lower-treatment-scores condition at the *p* < 0.01 level of significance. Turning to the average length of comments associated with the criteria-specific and nonspecific topics, we find that whereas the average length of criteria-specific comments was 31.8 words (s.d. = 33.43), the average length of nonspecific topics was 13.0 words (s.d. = 4.93), a significant difference of 18.8 words (*t*(293) = 4.61, *p* < 0.01).

Overall, this suggests that exposures to lower scores compelled evaluators to spend more effort processing the evaluative task at hand and, particularly, to identify and justify overlooked or underweighted weaknesses. This is consistent with the notion that negative information, or “critical scores,” attracts greater attention and requires more in-depth information processing (Ito et al. 1998).

## 6. Implications of Score-Updating Patterns by Reviewers

We now turn to the overall effect of the treatments on the distribution of proposal scores across both studies. To ensure the external validity of our results, we focused only on the cases where the *fabricated* other reviewers’ scores happened to match the *actual* other reviewers’ scores—that is, “true exposures.” Specifically, we included in the true-exposures subset any evaluator-proposal pairs where the mean of the other reviewers’ *actual* scores on the same proposal fell within the range of exogenously varied (“fabricated”) treatment scores. A total of 160 evaluation-proposal pairs (114 evaluators and 72 proposals) fit the inclusion criterion.

**Figure 5.**
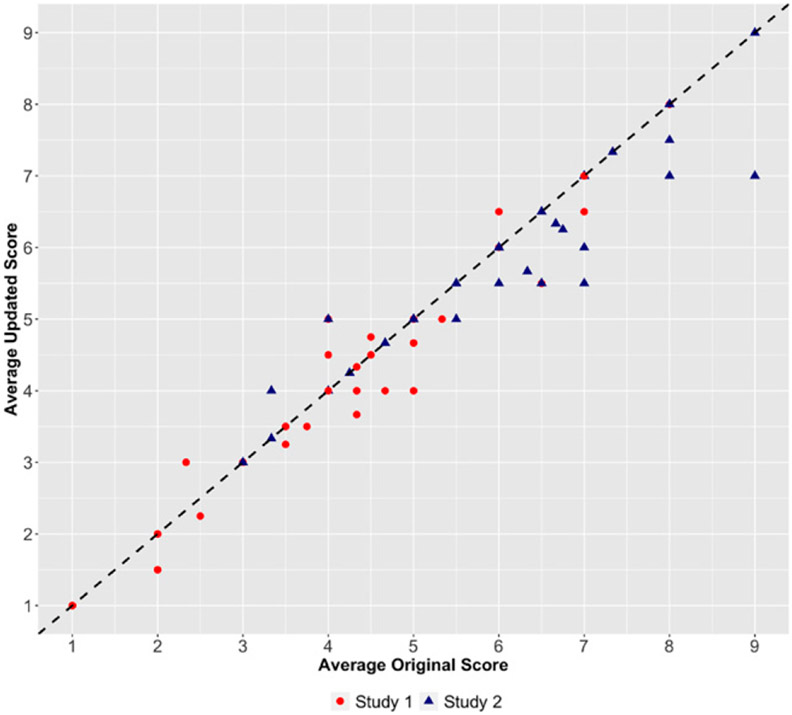
Scatter Plot of Average Updated (Postupdate) vs. Original (Preupdate) Scores

Figure 5 plots the average updated scores and the average original scores for each proposal, and the dashed black line is the 45-degree line representing no change. We find that in this subset, the score treatments caused updated scores to become systemically more critical. Of 160 evaluator-proposal pairs, 40 (25%) evaluation scores decreased, 14 (8.75%) increased, and 106 (66.25%) remained the same, with evaluators being about 2.9 times more likely to lower than raise their scores. Also noteworthy is that the valenced treatment scores reduced the degree of noise in scoring (measured using standard deviation) in both studies: In study 1, the mean standard deviation decreased from 2.18 to 1.92, and in study 2, it decreased from 1.14 to 0.95.

**Figure 6.**
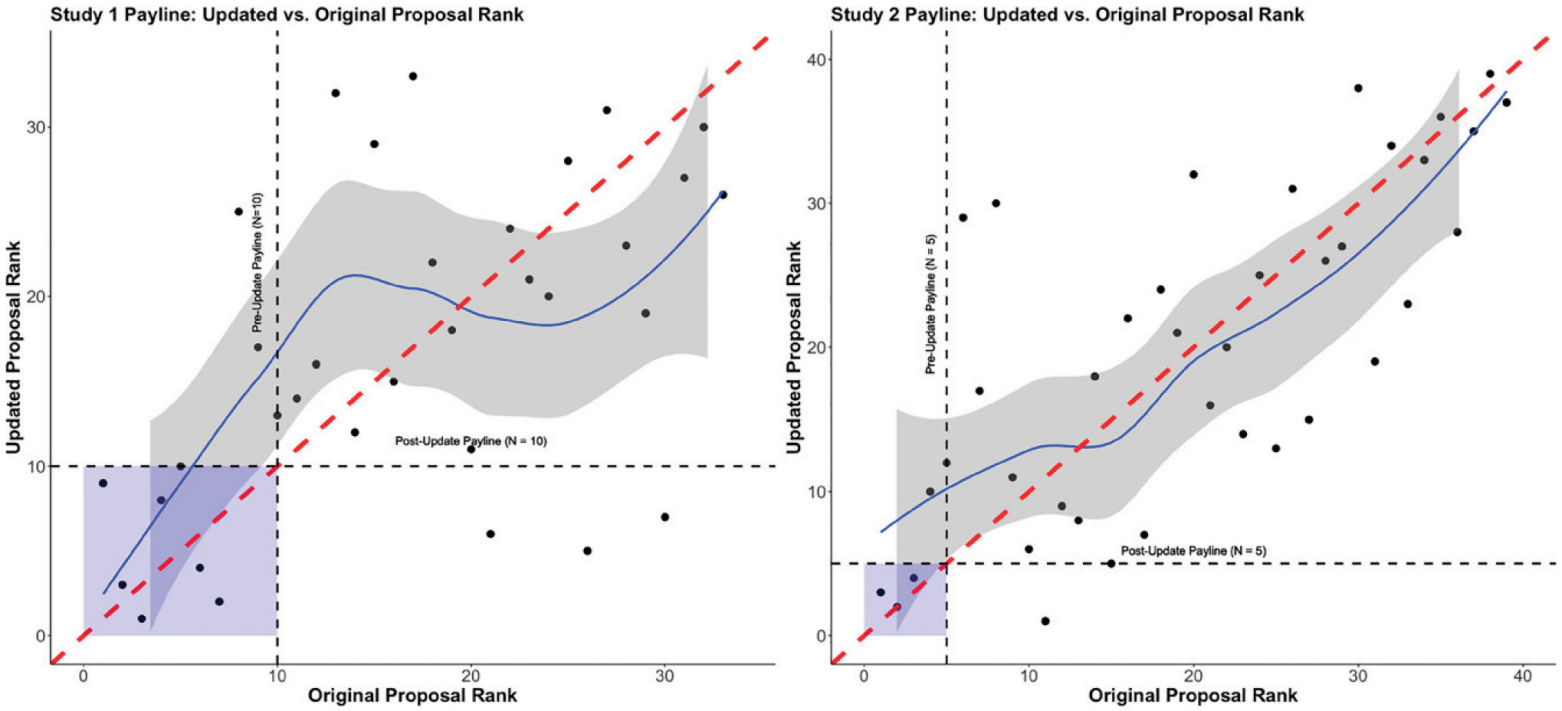
Comparison of Updated vs. Original Proposal Ranks for Study 1 (Left) and Study 2 (Right)

Are such changes in scores substantively important? To answer this question, we first rank-order the proposals on the mean original score (pretreatment) and then the mean updated score (posttreatment) and compare the correlation between the two rankings.^6^ Figure 6 presents two scatterplots of the updated versus original proposal ranks for study 1 (left; *n* = 33 proposals) and study 2 (right; *n* = 39 proposals), respectively, as well as two other indicators: The red dashed line is the 45-degree line representing no turnover in proposal ranking, and the black dashed lines are the paylines (vertical = original/preupdate payline; horizontal = postupdate payline). The paylines correspond to the number of awarded proposals in each study (i.e., 10 in study 1 and five in study 2). First, we observe that both plots are very noisy, with few points falling on the 45-degree line, even though the correlation is moderate (study 1: *ρ* = 0.505; study 2: *ρ* = 0.733). This suggests that there is significant turnover in the proposal rankings before and after the exposures to treatment scores—that is, initially highly ranked proposals may lose out if the reviewers are exposed to scores from others.^7^ Second, if we focus on the “highest-quality” proposals that fall within the payline in each study, represented by the blue shaded region, we observe that in study 1, six of the original 10 proposals would still be funded postupdate, whereas in study 2, three of the original five proposals would still be funded postupdate—corresponding to a sizeable turnover rate of 40% in both studies.

**Figure 7.**
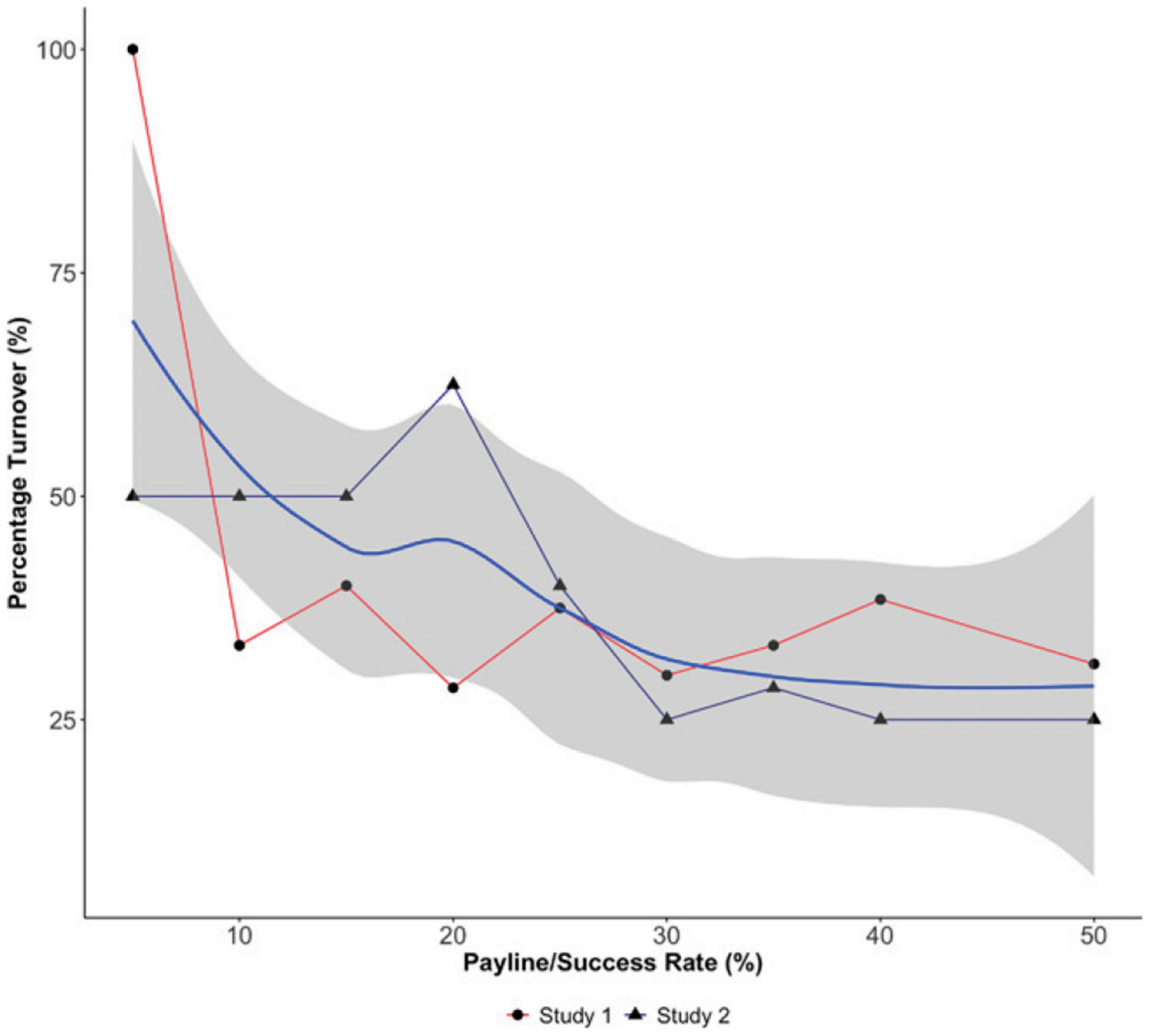
Percentage Turnover in Winning Proposals as a Function of the Payline (Success Rate)

Next, we ask how the turnover percentage in awarded proposals would have varied as a function of “hypothetical paylines,” ranging from 5% to 50%. Figure 7 shows the turnover percentages after exposures to other reviewers’ opinions, showing that they can have significant implications on funding-allocation decisions, even for quite generous paylines, as indicated by the smoothed locally estimated scatterplot smoothing fitted line.

## 7. Discussion

A fundamental challenge for senior executives and research administrators is how to ensure that their innovation pipelines focus on the highest-quality projects (Powell and Snellman 2004, Boudreau et al. 2016). The process of evaluating and selecting novel projects and ideas is, thus, a critical and challenging part of innovation (Li and Agha 2015, Criscuolo et al. 2017). Evaluations by experts are often considered the gold-standard method of assessing project quality (Chubin and Hackett 1990). This said, although expert-evaluation processes are widely used and take many forms, the implications of how evaluations are designed on which projects win and lose are poorly understood. This knowledge gap is a particularly important one because, unlike the reallocation of substantial sums of funding, the design of evaluation processes is relatively actionable, and the choices may rest with just one administrator (Azoulay and Li 2020).

### 7.1. Results Summary and Contributions to Literature

Our objective was to understand the workings and implications of information sharing among evaluators. We focus on academic science because peer review lies at the heart of scientific discovery and the advancement of knowledge (Lamont 2009, Stephan 2012), but analogous evaluations are found economy-wide (Criscuolo et al. 2017) and are key to incumbent company strategy. Drawing on the literature on negativity bias, we exogenously varied the valence of the other reviewers’ scores. Using quantitative and qualitative measures, we found a clear and reproducible pattern: Negative information had a much stronger effect on people’s attention, information processing, and behavior, consistent with the negativity bias found in other domains (Baumeister et al. 2001, Rozin and Royzman 2001). Qualitative comments accompanying the evaluators’ decisions to adjust their scores suggest that, as a result of exposures to critical information, evaluators devoted greater attention to evaluation-criteria-specific tasks, such as scrutinizing the proposal for critiques and weaknesses. In contrast, exposures to neutral and higher scores led to shorter comments, with a greater focus on non-criteria-specific aspects of evaluation, such as confidence in their judgment or achieving consistency with the other reviewers, which did not prompt additional information processing of the evaluation task at hand.

Thus, provided with the opportunity to deliberate and influence each other, evaluators are more likely to focus on proposal weaknesses than strengths. This asymmetry suggests that reviewers are more concerned with false positives (i.e., type I errors) than false negatives (i.e., type II errors), as the furnishing of negative information weighs more heavily on reviewers’ decisions than positive information of comparative magnitude. This finding may help explain what many see as “conservatism bias” in funding novel projects, which has conjured slogans such as “conform and be funded” and “bias against novelty” (Nicholson and Ioannidis 2012, Boudreau et al. 2016). If the risk of proposals is associated with their weaknesses, then, relative to independent evaluations, postsharing evaluations favor more conservative projects. These decisions, in turn, directly shape the disruptiveness of innovation occurring at the knowledge frontier.

This result departs significantly from the policy levers typically considered in stimulating high-risk, high-reward research. In practice, governments, foundations, and companies have generally responded to the perceived conservatism bias by allocating funds designated for risky projects (Heinze 2008, Gewin 2012). Meanwhile, the (relatively inexpensive) changes to the evaluation process have received less consideration and much less experimentation. Our work shows that small changes to the format of the evaluation process not only change the rank order and selection of winning proposals, but may also change the conservatism of the selections.

### 7.2. Directions for Future Research

Our research points to several directions for future work. First, researchers and scientific administrators should investigate the link between evaluation format and conservatism more directly. Our work did not measure project risk directly, nor did it track long-term outcomes.

Second, future work can explore whether our findings are sensitive to other forms of information exposure. Also, to insert exogenous variation into the process, we exposed evaluators to artificial scores from other reviewers, but a logical next step would be to examine how evaluators’ behaviors would change if they were exposed to actual scores and critiques of strengths and weaknesses.

Third, our experimental setting represents a trade-off between depth and generalizability of our findings. Although we found that our experiment replicated across two settings, both studies were conducted in the field of biomedicine, which entails substantial collaboration and interdisciplinary research (Jones et al. 2008, Leahey et al. 2017). Because norms for peer evaluation and collaboration versus competition vary across fields (Thursby et al. 2018), further work could aim to extend these findings to more settings.

Lastly, a complementary approach would be to further explore the cognitive process of reviewing and train evaluators to evaluate strengths and weaknesses more symmetrically. Some work suggests that, although evaluators tend to agree more on the relative weaknesses of proposed work, they are less effective at identifying its strengths (Cicchetti 1991). Overall, these directions represent fruitful avenues for improving evaluation and selection processes in science and innovation more broadly.

## Supplementary Material

Online Appendix

## Figures and Tables

**Table 1.** Summary Statistics of Count of Proposals Reviewed by Evaluator

	Treatment			Control		
Statistic	Study 1	Study 2	Pooled	Study 1	Study 2	Pooled
No. of evaluators	244	89	333	34	3	37
Mean (s.d.)	1.59 (1.05)	3.75 (2.44)	2.17 (1.82)	1.00 (0.00)	1.33 (0.58)	1.03 (0.16)
Min, max	1, 6	1, 8	1, 8	1, 1	1, 2	1, 2
No. of pairs	389	334	723	34	4	38

**Table 2.** Distribution of Treatment Scores Valence by Original Score

Original score	Study 1		Study 2		
	Lower	Higher	Lower	Neutral	Higher
1	0	28	0	1	0
2	0	38	0	6	3
3	30	34	0	9	23
4	29	34	12	15	10
5	32	38	21	15	12
6	43	34	24	27	20
7	44	0	61	24	0
8	5	0	15	23	0
9	—	—	8	5	0

**Table 3.** Covariate Balance Check by Treatment-Score Valence (*N* = 723)

Variable	Study 1 (*N* = 389)	Study 2 (*N* = 334)
*Female*	*χ*^2^(1,*N* = 389) = 0.362, *p* = 0.547	*χ*^2^(2,*N* = 334) = 1.026, *p* = 0.599
*High rank*	*χ*^2^(2,*N* = 389) = 0.992, *p* = 0.319	*χ*^2^(2,*N* = 334) = 3.116, *p* = 0.211
*Intellectual distance*	*χ*^2^(1,*N* = 389) = 1.447, *p* = 0.229	*χ*^2^(2,*N* = 334) = 0.149, *p* = 0.928
*Expertise*	*F*(1,387) = 0.050, *p* = 0.817	*F*(2,330) = 0.800, *p* = 0.450
*Original score*	*F*(1,387) = 116.92, *p* = 0.000	*F*(2,331) = 43.77, *p* = 0.000

**Table 4.** Correlation Table of Main Variables (*N* = 723)

Variable	Mean	s.d.	Min	Max	1	2	3	4	5	6
1. *Change in score*	−0.145	0.880	−4	4	1.000					
2. *Treatment scores valence*	1.068	0.909	0	2	−0.626	1.000				
3. *Intellectual dist.*	0.530	0.499	0	1	0.005	−0.043	1.000			
4. *Expertise*	3.374	0.943	1	5	−0.025	−0.014	0.020	1.000		
5. *Female*	0.350	0.477	0	1	−0.001	0.015	0.016	−0.033	1.000	
6. *Tenured*	0.536	0.499	0	1	0.051	−0.056	−0.013	0.036	−0.207	1.000
7. *Original score*	5.057	1.906	1	9	−0.340	0.482	0.019	−0.105	0.065	−0.060

**Table 5.** Estimated Relationships Between Change in Evaluation Score and Treatment Scores Valence

Variable	Full sample (all scores)				Restricted sample (middle scores)			
	Model 1: Treatment scores	Model 2: Covariates	Model 3: Original score FE	Model 4: Evaluator FE	Model 5: Treatment scores	Model 6: Covariates	Model 7: Original score FE	Model 8: Evaluator FE
Randomized: Baseline = neutral treatment scores								
Lower scores	−0.759*** (0.0550)	−0.756*** (0.0557)	−0.753*** (0.0580)	−0.867*** (0.103)	−0.625*** (0.0816)	−0.618*** (0.0827)	−0.663*** (0.0823)	−0.911*** (0.158)
Higher scores	0.449*** (0.0499)	0.452*** (0.0523)	0.434*** (0.0581)	0.527*** (0.106)	0.561*** (0.0635)	0.567*** (0.0650)	0.503*** (0.0701)	0.512*** (0.157)
Intellectual distance		−0.0380 (0.0518)	−0.0297 (0.0508)	0.00304 (0.0696)		−0.0293 (0.0712)	−0.0180 (0.0674)	−0.00965 (0.0959)
Covariates								
Expertise		−0.0237 (0.0260)	−0.0360 (0.0254)	−0.00824 (0.0566)		−0.0216 (0.0313)	−0.0464 (0.0291)	0.00878 (0.0636)
Female		0.0246 (0.0554)	0.0371 (0.0549)			−0.0126 (0.0740)	0.00868 (0.0718)	
Tenured		0.0411 (0.0540)	0.0540 (0.0529)			0.0498 (0.0690)	0.0526 (0.0688)	
Constant	0.0242 (0.0265)	0.0909 (0.0923)	−0.180 (0.124)	−0.407 (0.248)	−0.0466 (0.0394)	0.0138 (0.114)	0.413*** (0.137)	0.150 (0.268)
Original Score FE	N	N	Y	Y	N	N	Y	Y
Evaluator FE	N	N	N	Y	N	N	N	Y
Observations	723	722	722	544	430	430	430	305
*R* ^2^	0.433	0.433	0.452	0.461	0.425	0.426	0.466	0.254
No. of proposals	97	97	97	94	95	95	95	82
No. of evaluators	333	333	333	155	266	266	266	141

*Notes.* Sample size drops from 723 to 722 in model 2 because of missing expertise in one evaluator-proposal pair from study 2. Robust standard errors are in parentheses.

*** *p* < 0.01.

**Table 6.** Overview of Qualitative Data Taxonomy and Coding for Study 2

Primary topic	Axial code	Open code examples
Criteria-specific	Impact	“Proposal has minimal impact if any.” “Highly ambitious, well thought out, with interesting translation potential.”
	Design and methods	“Lacks description of study participants, data analyses section and etc.” “Not especially well designed but data worth having.”
	Feasibility	“Limited information about the feasibility of such a study.” “… I’m also somewhat concerned about recruitment and specimen collection in the time allotted for the project.”
	Novelty	“Not so original.” “There are many published and ongoing studies addressing circadian misalignment and microbiome. The novelty of this study is limited.”
	Reevaluate proposal	“I was between a 3 and a 4. In reviewing the grant again, a 3 would be appropriate.” “Reconsidered.”
	Overall assessment	“Good bioinformatics application.” “Very good proposal utilizing a great cohort.”
Nonspecific	Consistent with others	“My score is within the range.” “Upon reviewing the other applications, I agree with the other reviewers.”
	Lack of expertise	“I attribute my original score to lack of expertise. Changed to reflect enthusiasm of other reviewers.” “Changed score because this is not an area I know.”
	Review process	“I realize I was using a higher bar than is optimal for a pilot grant.” “First reviewed grant and felt there were several weaknesses but was unsure how to grade.”
	Confident in judgment	“I am confident that my judgment is fair.” “I still rate it as a 5.”

## Footnotes

1 We do not focus on this intellectual distance manipulation, as the domain cues were difficult to make salient in the online setting, and the manipulation produced no measurable effects.

2 We note that the sponsoring organization only took the original scores, and not the updated scores, for the awards.

3 We omitted the evaluator’s self-reported *Confidence* because it was not consistently collected in both studies.

4 To directly compare the magnitude of the score updates between exposures to lower and higher treatment scores, we estimate Equation (1), but replace the *Change in evaluation score* with the *Absolute change in evaluation score.* The results in Table A4 in the online appendix indicate that the coefficient for lower treatment scores is statistically different from higher treatment scores (model 1: 0.246, s.e. = 0.0573) and remains statistically significant in the full model with original score FE (model 3: 0.106, s.e. = 0.0618).

5 The evaluation form asked evaluators to provide a subscore about the potential impact of the proposed work, where *Impact* was defined as “having potential translational benefit to patients or physicians in terms of improved treatments or an increased understanding of disease.”

6 The original proposal rank is calculated using the average across all original scores, whereas the updated proposal rank is restricted to the subset described above.

7 Recall that the award administrators used the original scores to determine funding decisions.
