# Supplementary material for: Conservatism Gets Funded? A Field Experiment on the Role of Negative Information in Novel Project Evaluation
Source: Manage Sci. Author manuscript; Available in PMC 2022 Oct 4. (PMC9531843; doi:10.1287/mnsc.2021.4107)

Appendix for

When Do Experts Listen To Other Experts?
The Role of Negative Information in Evaluations of Novel Projects

Table A1. Distribution of Number of Evaluators Per Proposal By Study and Conditions

	Treatment			Control		
	Study 1	Study 2	Pooled	Study 1	Study 2	Pooled
# of proposals	47	50	97	25	2	27
Mean (s.d.)	8.28 (1.35)	6.68 (2.59)	7.45 (2.22)	1.36 (0.91)	2.00 (0.00)	1.41 (0.89)
Min, Max	5, 10	3, 13	3, 13	1, 5	2, 2	1, 5
# pairs	389	334	723	34	4	38

Table A2. Treatment Score Valences for Study 1

Original Score	Valence	Treatment Score Ranges
1	Positive	3-6
2	Positive	4-7
3	Negative	1-3
3	Positive	5-7
4	Negative	1-3
4	Positive	6-8
5	Negative	1-4
5	Positive	7-8
6	Negative	1-4
6	Positive	7-8
7	Negative	2-5
8	Negative	3-6

Table A3. Number of Evaluator-Proposal Pairs By Treatment Scores and Intellectual Distance Condition

Study 1	Intellectual Distance Condition		
Information Condition	Close	Distant	N
Control	--	--	34
Higher Scores	91	115	206
Lower Scores	92	91	183
Study 1 Total	183	206	423
**Study 2**	**Intellectual Distance Condition**		
Information Condition	Close	Distant	N
Control	--	--	4
Low Scores	47	53	101
Moderate Scores	59	64	123
High Scores	50	60	110
Study 2 Total	156	177	338

Table A4. Absolute Change in Evaluation Score on Treatment Scores Valence (Full Sample)

	Dependent Variable: Absolute Change in Evaluation Score			
VARIABLES	Model 1<br>Treatment scores	Model 2<br>Evaluator attributes	Model 3<br>Original score FE	Model 4<br>Evaluator FE
**Randomized**				
Baseline = Higher treatment scores				
Lower treatment scores	0.246***	0.241***	0.106*	0.225**
	(0.0573)	(0.0557)	(0.0618)	(0.0990)
Neutral treatment scores	-0.416***	-0.447***	-0.542***	-0.612***
	(0.0447)	(0.0470)	(0.0554)	(0.0994)
Intellectual distance		-0.0190	-0.0190	-0.0268
		(0.0492)	(0.0476)	(0.0686)
**Covariates**				
Expertise		-0.110***	-0.0937***	-0.0972*
		(0.0274)	(0.0272)	(0.0511)
Female		0.0345	0.0202	
		(0.0484)	(0.0485)	
Tenured		0.00312	0.00701	
		(0.0553)	(0.0531)	
Constant	0.494***	0.870***	0.498***	0.656***
	(0.0345)	(0.107)	(0.123)	(0.220)
Original Score FE	N	N	Y	Y
Evaluator FE	N	N	N	Y
Observations	723	722	722	544
R-squared	0.125	0.152	0.155	0.212
Number of proposals	97	97	97	94
Number of evaluators	334	333	333	155

Robust standard errors in parentheses; *** p<0.01, ** p<0.05, * p<0.1

Table A5. Absolute Change in Evaluation Score on Treatment Scores Valence (Restricted Sample of Middle Scores)

	Dependent Variable: Absolute Change in Evaluation Score			
VARIABLES	Model 1 Treatment scores	Model 2 Evaluator attributes	Model 3 Original score FE	Model 4 Evaluator FE
**Randomized**				
Baseline = Higher treatment scores				
Lower treatment scores	0.125*	0.118*	0.113*	0.305**
	(0.0744)	(0.0690)	(0.0678)	(0.123)
Neutral treatment scores	-0.540***	-0.566***	-0.601***	-0.538***
	(0.0635)	(0.0612)	(0.0620)	(0.138)
Intellectual distance		-0.101*	-0.104*	-0.0841
		(0.0568)	(0.0570)	(0.0935)
**Covariates**				
Expertise		-0.111***	-0.0979***	-0.146**
		(0.0279)	(0.0275)	(0.0586)
Female		-0.00602	-0.0248	
		(0.0627)	(0.0635)	
Tenured		-0.0265	-0.0280	
		(0.0621)	(0.0611)	
Constant	0.567***	1.015***	0.765***	0.817***
	(0.0475)	(0.119)	(0.136)	(0.215)
Original Score FE	N	N	Y	Y
Evaluator FE	N	N	N	Y
Observations	430	430	430	305
R-squared	0.130	0.147	0.151	0.254
Number of proposals	95	95	95	82
Number of evaluators	266	266	266	141

Robust standard errors in parentheses; *** p<0.01, ** p<0.05, * p<0.1

Table A6. Estimated Relationships Between Change in Evaluation Score and Treatment Score Valence (Study 1 only)

	Dependent Variable: Change in Evaluation Score			
VARIABLES	Model 1<br>Treatment scores	Model 2<br>Covariates	Model 3<br>Original Score FE	Model 4<br>Evaluator FE
**Randomized**				
Baseline = Higher treatment scores				
Lower treatment scores	-1.044***	-1.046***	-1.074***	-1.213***
	(0.0683)	(0.0684)	(0.0811)	(0.133)
Intellectual distance		-0.0289	-0.00454	0.0764
		(0.0604)	(0.0566)	(0.0942)
**Covariates**				
Expertise		0.0106	-0.00496	0.0510
		(0.0304)	(0.0284)	(0.0665)
Female		0.0222	0.0578	
		(0.0656)	(0.0602)	
Tenured		0.00304	0.0298	
		(0.0641)	(0.0582)	
Constant	0.428***	0.397***	0.124	-0.126
	(0.0423)	(0.138)	(0.135)	(0.272)
Original score FE	N	N	Y	Y
Evaluator FE	N	N	N	Y
Observations	389	389	389	224
R-squared	0.541	0.540	0.560	0.579
Number of evaluators	244	244	244	79
Number of proposals	47	47	47	39

Note: In study 1, evaluators were only exposed to lower or higher scores (i.e., no neutral scores).
Robust standard errors in parentheses; *** p<0.01, ** p<0.05, * p<0.1

Table A7. Estimated Relationships Between Change in Evaluation Score and Treatment Score Valence (Study 2 only)

	Dependent Variable: Change in Evaluation Score			
VARIABLES	Model 1<br>Treatment scores	Model 2<br>Covariates	Model 3<br>Original Score FE	Model 4<br>Evaluator FE
**Randomized**				
Baseline = Neutral treatment scores				
Lower treatment scores	-0.911***	-0.899***	-0.892***	-0.950***
	(0.0927)	(0.0910)	(0.0956)	(0.116)
Higher treatment scores	0.613***	0.616***	0.636***	0.693***
	(0.105)	(0.105)	(0.0986)	(0.128)
Intellectual distance		-0.0526	-0.0583	-0.0432
		(0.0862)	(0.0881)	(0.0991)
**Covariates**				
Expertise		-0.0784	-0.0843*	-0.0601
		(0.0481)	(0.0500)	(0.0968)
Female		0.0235	0.0214	
		(0.0946)	(0.0958)	
Tenured		0.0633	0.0496	
		(0.0906)	(0.0909)	
Constant	0.0242	0.260*	0.337*	-0.0538
	(0.0267)	(0.149)	(0.200)	(0.366)
Original Score FE	N	N	Y	Y
Evaluator FE	N	N	N	Y
Observations	334	333	333	320
R-squared	0.364	0.375	0.391	0.428
Number of proposals	50	50	50	50
Number of evaluators	89	89	89	76

Note: Sample size reduces by one in Model 2 due to missing expertise for one evaluator-proposal pair.
Robust standard errors in parentheses; *** p<0.01, ** p<0.05, * p<0.1

Table A8. Change in Evaluation Score and Treatment Scores Valence with Control (Full Sample)

	Dependent Variable: Change in Evaluation Score			
VARIABLES	Model 1<br>Treatment scores	Model 2<br>Covariates	Model 3<br>Original Score FE	Model 4<br>Evaluator FE
**Randomized**				
Baseline = Neutral treatment scores				
Lower treatment scores	-0.760***	-0.756***	-0.753***	-0.882***
	(0.0591)	(0.0608)	(0.0617)	(0.106)
Higher treatment scores	0.447***	0.451***	0.434***	0.559***
	(0.0431)	(0.0476)	(0.0589)	(0.113)
Control	-0.0283	0.0139	0.0748	
	(0.0273)	(0.0475)	(0.0753)	
Intellectual distance		-0.0383	-0.0298	-0.0157
		(0.0552)	(0.0533)	(0.0714)
**Covariates**				
Expertise		-0.0244	-0.0359	-0.0121
		(0.0291)	(0.0282)	(0.0598)
Female		0.0247	0.0371	
		(0.0526)	(0.0509)	
Tenured		0.0408	0.0540	
		(0.0604)	(0.0608)	
Constant	0.0248	0.0941	-0.181	-0.192
	(0.0271)	(0.101)	(0.119)	(0.261)
Original Score FE	N	N	Y	Y
Evaluator FE	N	N	N	Y
Observations	757	726	726	564
R-squared	0.433	0.433	0.453	0.468
Number of proposals	97	97	97	94
Number of evaluators	365	336	336	204

Robust standard errors in parentheses; *** p<0.01, ** p<0.05, * p<0.1

Figure A1. Screenshots of Evaluation Criteria and Sample Treatment from Study 2 (Study 1 has similar design and presentation)

Proposal XX

Dear Reviewer:

Thank you for agreeing to assist us with the review process for the [redacted] **Microbiome Pilot Grant Opportunity**.

The objective of this RFA was to solicit proposals that will promote a greater understanding of the role(s) microbiomes play in maintenance of normal human physiology and in the manifestation and treatment of human disease. There was no restriction on the area of human health to be investigated in the proposal. Applicants were encouraged to think broadly about the interactions between microbiomes and human physiology and ecology in formulating their proposals.

You can read more about the opportunity by clicking here.

Note: You will be able to access the proposal and review form after entering your information.

First Name

Last Name

How do you characterize your primary disciplinary expertise for the purposes of this review?

- Microbiome related
- Disease specific
- Other, please specify

Next

Please review this Proposal_AD_1 . Then, complete each of the following questions. You may save your progress and return to this review at any time before the review deadline.

1. How would you assess your **expertise** on the topic the application, **XX**, addresses?

2. If successful, what is the level of **impact** of the proposed work? **Impact** can be defined here as having potential translational benefit to patients or physicians in terms of improved treatments or an increased understanding of disease.

3. How **innovative** is the proposal? **Innovative** can be defined here as likely to lead to a new technology or new knowledge, the unanticipated application of an existing technology or concept, or a novel approach that enhances an established modality or concept.

4. As described in **Proposal XX** is the project **feasible**? **Feasible** can be defined here as achievable, within the year of support, by following the suggested research plan.

5. As proposed, does the project address an important clinical and translational medicine question?

6. As proposed, is the project likely to develop sufficient proof of concept information such that the team can proceed to look for additional funding by the end of this pilot grant (e.g. submit a grant using preliminary data generated with the pilot funding)?

7. If you have reviewed at least three proposals for this RFA, would you rate this proposal among the top three?

8. Please provide an **overall scientific merit score** to this application, using a scale from 1 to 9, where 1 is exceptional, and 9 is poor.

<< Next

Although we lacked the capacity to conduct ***in-person review panels*** for this pilot grant opportunity**,** we would nevertheless like to let you know what other reviewers thought of this application. The reviewer pool included **microbiome and disease-specific experts**.

On the next page are the scores we have received from **microbiome experts**.

After seeing these scores you may update your overall score if you see fit.

Note: You *must* continue to the next screen in order to submit your review scores, regardless of whether you wish to update your score.

Continue

Attribute	Your Score	Range of other reviewers' scores
Overall Score	1 - Exceptional	1-3

If you would like to update your overall score of **1 - Exceptional** for proposal **xx**, please do so here:

Please explain

Submit

Figure A2. Distribution of Evaluation Score Updates (Study 1: left; Study 2: right)

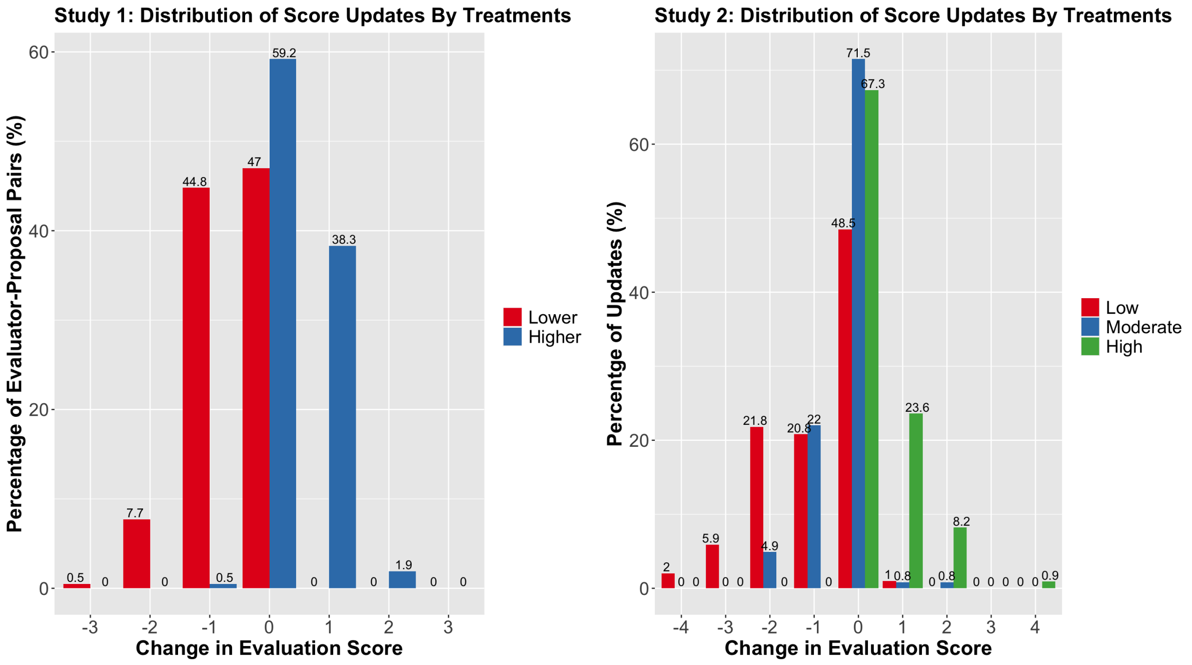

Supplement: Online Appendix [file NIHMS1798634-supplement-Online_Appendix.pdf]
